# Scale of adverse events associated to nursing practices: a psychometric study in Portuguese hospital context^*^

**DOI:** 10.1590/1518-8345.2595.3093

**Published:** 2018-11-29

**Authors:** Teresa Neves, Vitor Rodrigues, João Graveto, Pedro Parreira

**Affiliations:** 1 Centro Hospitalar e Universidade de Coimbra, Coimbra, Portugal.; 2 Faculdade de Medicina, Universidade de Coimbra, Coimbra, Portugal.; 3 Escola Superior de Enfermagem de Coimbra, Coimbra, Portugal.

**Keywords:** Patient Safety, Nursing Care, Safety Management, Health Care Quality, Access, and Evaluation, Psychometrics, Validation Studies

## Abstract

**Objective:**

to contribute to the validation study of the Scale of Adverse Events associated with Nursing Practices in the hospital context.

**Method:**

cross-sectional study, in public hospital units, in the central and northern regions of Portugal. The exploratory factor analysis of the Scale of Adverse Events associated to Nursing Practices was conducted with a sample of 165 nurses and the confirmatory factorial analysis was made with a sample of 685 nurses. Reliability, internal consistency and construct validity were estimated. The invariance of the model was evaluated in two subsamples to confirm the stability of the factorial solution.

**Results:**

the global sample consisted of 850 nurses aged between 22 and 59, mostly licensed professionals. The model had a good overall fit in the subscales (Nursing Practices: χ^2^/df = 2.88, CFI = 0.90, GFI = 0.86, RMSEA = 0.05, MECVI = 3.30; Adverse Events: χ^2^/df = 4.62, CFI = 0.93, GFI = 0.95, RMSEA = 0.07, MECVI = 0.39). There was a stable factor structure, indicating strong invariance in the subscale Nursing Practices and structural invariance in the subscale Adverse Events.

**Conclusion:**

the refined model of the Scale of Adverse Events associated with Nursing Practices revealed good fit and stability of the factorial solution. The instrument was adjusted to evaluate the perception of nurses about adverse events associated with health care, precisely nursing care, in the hospital setting.

## Introduction

Health care safety has become one of the priorities of national and international health organizations in recent decades. Scientific evidence indicates high rates of adverse events (AE) arising from health care provision, with an impact on patients’ health and economic-financial systems, being an important indicator of the safety of care measures. However, the reporting of adverse events is still incipient, making it difficult to estimate their impact^(^ ^1^ ^-^ ^3^ ^)^.

Health-related AE result from a succession of occurrences that favor unexpected/unwanted events arising from health care interventions due to failure or omission in its provision instead of factors associated with the patients’ underlying pathology. These can cause adverse effects/harm to the patients, including permanent damages or even death, influencing the increase in morbidity and mortality, hospitalization time and consequent associated costs, with an impact on the health systems^(^ ^4^ ^-^ ^5^ ^)^.

AE result from the combination of several factors in highly complex environments, including individual factors related to the patient, factors related to the health professionals such as professional skills, but also economic-financial constraints and institutional weaknesses such as insufficient human resources, overcrowding of patients, inadequate structure and equipment, misfit accommodation care, poor hygiene conditions, among others. There are also aspects related to the work environment, safety culture, leadership style and structure and development of the care process as determinants of health care safety^(^ ^1^ ^-^ ^2^ ^,^ ^6^ ^-^ ^8^ ^)^.

The development of indicators and management support instruments for the measurement of care quality and safety is essential to minimize the risks associated with health care, supporting the decision-making process with a view to continuous improvement. This is particularly relevant in hospital settings, and nurses have a crucial role in the identification and management of AE through direct and systematic interventions to patients ^(^ ^9^ ^)^.

The Scale of Adverse Events associated with Nursing Practices (SAEANP) emerges as an instrument for the diagnosis and monitoring of the frequency of safety-related processes/practices and the subsequent result of risk and occurrence of AE. The scale evaluates different AE associated with hospital nursing care in a cross-sectional way, namely, deficits of surveillance, clinical judgment and patient advocacy, falls, pressure ulcers, medication errors and healthcare-associated infections (HAIs)^(^ ^4^ ^)^.

However, the initial exploratory factor analysis (EFA) developed by the authors of the scale resulted in a factorial solution slightly different from the predicted, evident mainly in the subscale of “risk perception and occurrence of AE”, due to the absence of homogeneity in the criterion of grouping of items according to dimensions. In some dimensions, grouping by type of AE was verified, with association between perception of risk and occurrence. However, with regard to falls and pressure ulcers, the perception of risk is isolated from the perception of occurrence. It was also evidenced the need to remove some items from the original scale, and the suggestion to include new items and restructure previous items. It was then proposed the development of a revised version of the scale, inciting the need for new psychometric evaluation studies^(^ ^4^ ^)^.

In this context, given the scarcity of instruments for evaluation of adverse events associated with nursing practices, it is fundamental to evaluate the factorial structure and the invariance of measurement of this instrument, given the importance of obtaining valid and reliable instruments with external and internal validity. The study is of decisive importance given the high potential of the SAEANP to monitor the nurses’ perception of AE, taking the instrument as a reference to evaluate the quality of nursing care.

Thus, the present study aims to contribute to the validation of the SAEANP in the hospital context.

## Method

A cross-sectional study was carried out to evaluate the psychometric properties of the SAEANP in 12 public hospital units in the central and northern regions of Portugal.

The target population includes nurses who perform functions in the provision of direct care to patients in 71 hospitalization, general surgery, internal medicine and orthopedic services of the hospitals studied.

As inclusion criterion in the sample, only nurses who provide direct nursing care were included. Nurses with management roles (“nurse managers”) were excluded.

Data collection took place between January 15^th^ and September 15^th^, 2015.

The sample size was calculated based on the objectives of the study, considering the need for the development of EFA and confirmatory factor analysis (CFA). A sample of 165 individuals was considered for the EFA, taking into account a ratio of three observations per variable^(^ ^10^ ^)^. In the case of the CFA, the sample size was based on a formula for the analysis of structural equations^(^ ^11^ ^)^, obtaining an estimate of 151 individuals. However, because the objective was to perform a psychometric evaluation, the selected sample consisted of the maximum number of participants in the target population, i.e. 685 nurses, to ensure the external validity of the results and the generalization of the conclusions for the population under study.

The data collection instrument was delivered personally to the nurse manager (who had the mediating role in the delivery and collection of questionnaires) of each service, who passed in to all nurses. The instrument was filled according to availability and then delivered in a sealed envelope.

The self-completed instrument includes socio-demographic questions and the revised SAEANP, after an initial evaluation of the psychometric properties, consisting of 55 items^(^ ^4^ ^,^ ^12^ ^)^. This is composed of two independent subscales, with process and result indicators, respectively, nursing practices (NP) and AE. The items are answered in a Likert-type scale of five points, where the score (1) corresponds to “Never” and the score (5) to “Always”.

**Figure 1:**
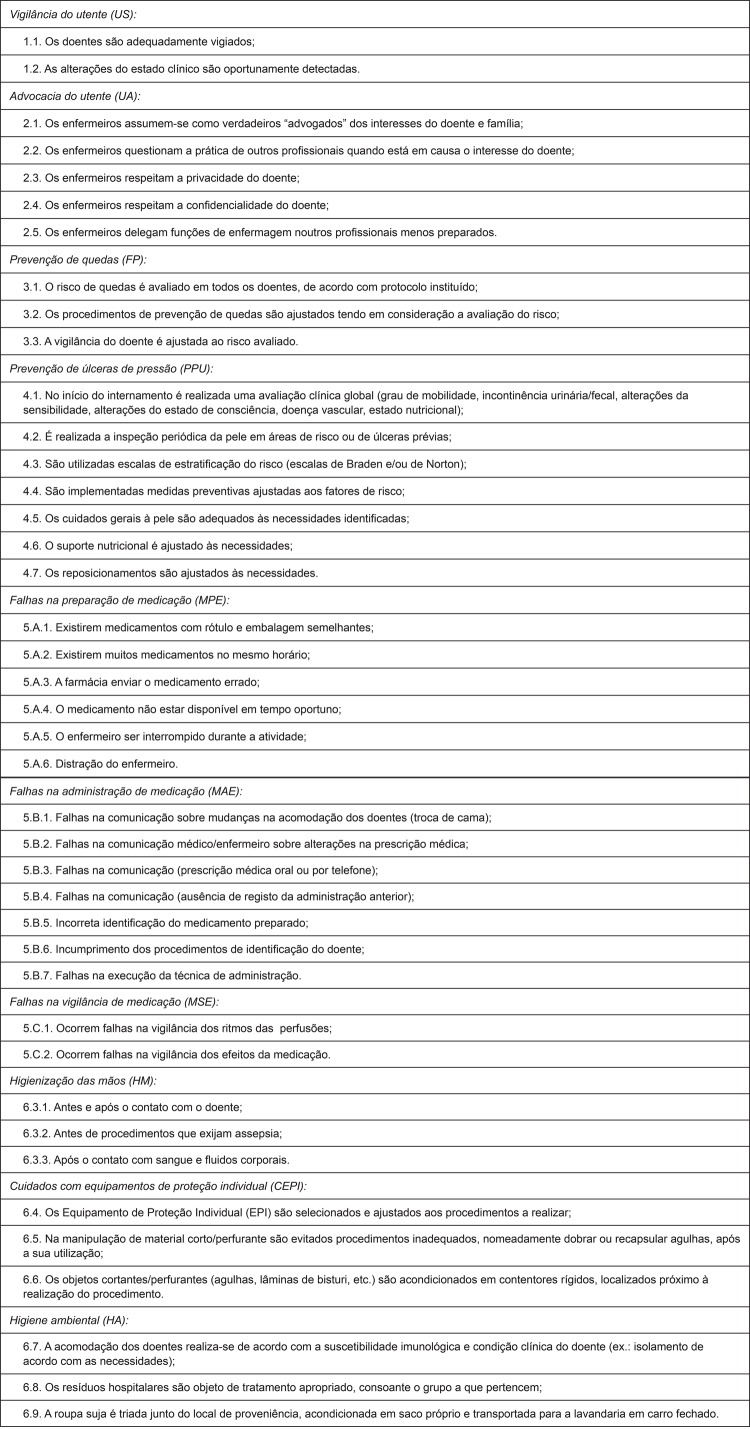
Scale of adverse events associated with nursing practices, Nursing Practices Subscale: revised version

The revised version of the NP subscale (41 items) integrates two new items to evaluate the fulfillment of preventive practices and failures in the application of professional norms, considering the original 10 dimensions, according to Figure 1 ^(^ ^4^ ^)^.

**Figure 2:**
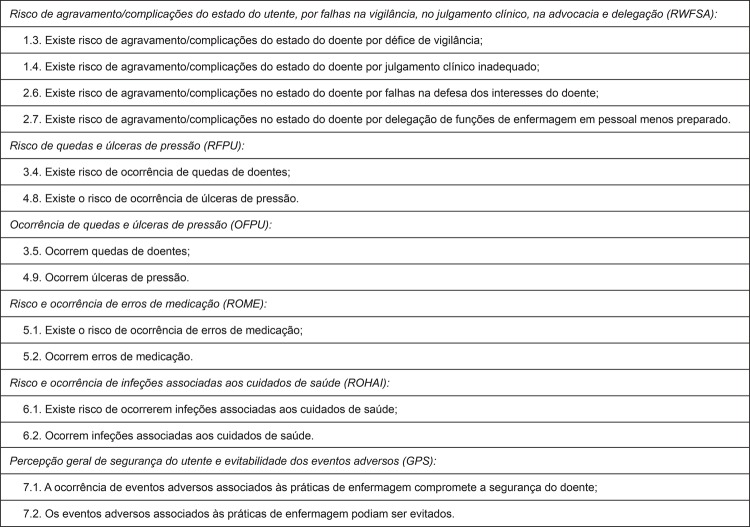
Scale of adverse events associated with nursing practices, Adverse Events Subscale: revised version

In the AE subscale (14 items), a new item was included, considering six dimensions, according to Figure 2 ^(^ ^4^ ^)^.

Given the results and suggestions of the previous study^(^ ^4^ ^)^, it was decided to perform the EFA of the revised version to evaluate the relational structure of the items of the two subscales. This was performed on the matrix of correlations, with extraction of the factors by the principal component method, followed by Varimax rotation. The factors with eigenvalue greater than one were retained, in agreement with Scree Plot and the percentage of retained variance, because the combination of several criteria avoids the retention of more or fewer factors than those relevant to the description of the latent structure^(^ ^13^ ^)^.

In a second phase of the study, we performed the CFA and invariance analysis to verify the adequacy of the data to the model under study.

Adherence to the normal distribution of variables was determined by the asymmetry (Sk) and kurtosis (Ku) coefficients, considering that |Sk| <3 and |Ku| <10 did not indicate significant deviations from the normal distribution, which impedes the analysis by the method of maximum likelihood. The presence of outliers was evaluated by the Mahalanobis’ square distance (D^2^). Omitted values were replaced by the mean of the series due to the small percentage in the sample (less than 3%)^(^ ^14^ ^)^.

The quality of the overall goodness-of-fit was evaluated according to different indices, considering acceptable values of χ^2^/df < 5, values of CFI and GFI > 0.90, RMSEA < 0.08, where the lowest MECVI indicates the model with the best external validity^(^ ^14^ ^-^ ^16^ ^)^. The modifications introduced to fit the model were supported by the modification indices (MI) (MI > 11; p < 0.001) produced by the AMOS software as well as theoretical considerations^(^ ^14^ ^)^.

The stability of the solution of the obtained factorial model was evaluated by cross-validation, comparing the indices observed in the test sample with the indices obtained in another independent sample, extracted from the same population, through multi-group analysis. The total CFA sample was thus divided randomly into approximately two equal parts. The factorial invariance (configuration, metric and structural) of the model was tested in both groups by comparison of the free model with a constricted model, in which the factor loadings, intercepts, residuals and variances/covariance of the two groups were fixed. The statistical significance of the difference between the two models was determined by the chi square test^(^ ^14^ ^)^.

The reliability and internal consistency of the construct were evaluated by composite reliability (CR) and Cronbach’s alpha (α), considering values above 0.70. The validity of the construct was determined in three subcomponents: convergent validity, calculated by the average variance extracted (AVE) by each factor, considering values greater than 0.50^(^ ^14^ ^-^ ^15^ ^)^ as indicators of convergent validity; discriminant validity was evident when the AVE of each of two factors was equal to or greater than the square of the correlation between these factors; and factorial validity was assessed considering the standardized factor loadings (λ) and the individual reliability (λ^2^), being also indicators of the goodness of the local fit. Usually, λ above 0.50 and subsequently λ^2^ higher than 0.25^(14.17)^ are considered appropriate, but in the social sciences sometimes lower values are accepted^(^ ^18^ ^)^. In the initial SAEANP study, the authors proposed λ greater than 0.30^(^ ^4^ ^)^, an option that was maintained in this investigation.

The descriptive analysis (measures of central tendency, dispersion and frequency) and EFA were made using the Statistical Package for the Social Sciences (version 22.0, SPSS An IBM Company, Chicago, IL), and the CFA and invariance analysis were made using the AMOS software (version 22, An IBM Company, Chicago, IL).

This study is part of a broader investigation approved by the Board of Directors and Ethics Committees of the hospital institutions, as well as the Ethics Committee of the Faculty of Medicine of the University of Coimbra, Portugal (Proc. EC 100/2014). The participation of the nurses was voluntary. Informed consent was requested from the participants, and the compliance with ethical principles such as anonymity and confidentiality was ensured.

## Results

The total sample was composed of 850 nurses (165 nurses of the EFA and 685 nurses of the CFA) out of the 1844 questionnaires distributed (response rate of 46.10%).

The analysis of the sociodemographic characteristics reveals that the overall sample is predominantly female (n = 686, 81.86%), aged 22-59 years (M = 36.11, SD = 7.97). As for educational qualifications, the most common academic degree was the licentiate degree (n = 748; 89.05%), and 222 (27.07%) were identified as nurses with a specialization in nursing. The most prevalent job bond was individual work contract (n = 483, 59.70%), with a workload of 40 hours per week (n = 708, 86.03%) and work in shifts (n = 670, 81.71%).

Regarding the representativeness of the sample, the results of the chi-square test did not show significant differences between the study sample and the Portuguese nurses’ population^(^ ^19^ ^)^ (χ^2^ = 0.001, p = 0.978).

The descriptive analysis of the items shows that they present adequate psychometric sensitivity for the factorial analysis.

The sample adequacy test for EFA, in a sample of 165 nurses, showed good adequacy in the NP subscale (KMO = 0.84) and average adequacy in the AE subscale (KMO = 0.77). It was also concluded by the Bartlett’s test of sphericity that the variables are significantly correlated in both subscales (*p* < 0.001).

According to the rule of the eigenvalue superior to one and with the scree-plot, the relational structure of the NP subscale is explained by 11 latent factors (70.79% explained variance), while in the AE subscale is explained by five factors (69.20% of explained variance). However, considering the interpretation of the factorial solution, we chose maintaining a structure with six factors (74.66% explained variance). In addition, all commonalities are high (> 50%), considering the retained factors as appropriate to describe the latent correlational structure.

The NP subscale has an acceptable global internal consistency (α = 0.76); the emerging factors are close to the predicted theoretical dimensions, maintaining the following factors unchanged: “user surveillance” (US) (two items, α = 0.75), “fall prevention” (FP) (three items, α = 0.80), “prevention of pressure ulcers” (PPU) (seven items, α = 0.83), “medication preparation errors” (MPE) (six items, α = 0.84), “hand hygiene” (HH) (three items, α = 0.73), “care with personal protective equipment” (CPPE) (three items, α = 0.77) and “environmental hygiene” (EH) (three items, α = 0.79). The isolation of the factor “privacy and confidentiality” (PC) (α = 0.86), independently of the factor “user advocacy” (UA) (α = 0.60), both with two items, was evidenced. As for “medication administration errors” (MAE), a division was verified, giving rise to the “communication failure associated with medication administration” (CFAMA) factor, with four items (α = 0.83), while the remaining three items were grouped with the “medication surveillance errors” (MSE), resulting in the factor “failures in medication administration and monitoring” (FMAM), with five items (α = 0.88). The item 2.5 (*nurses delegate nursing functions to other less prepared professionals*) was eliminated by saturating the MPE factor, conditioning the interpretation.

The AE subscale has good internal consistency (α = 0.84), and the latent factors are translators of the theoretical dimensions. The “general perception of user safety and avoidance of adverse events” (GPS) factors (two items, α = 0.43), “risk and occurrence of medication errors” (ROME) (two items, α = 0.68) and “risk and occurrence of healthcare-associated infections” (ROHAI) (two items; α = 0.81) remained in line with the original structure. Regarding the “risk of worsening/complications of the patients’ state due to failures in surveillance**,** clinical judgment, advocacy and delegation” (RWFSA), this was divided in the factors “risk factors for worsening/complications of the patient’s condition due to failures in surveillance and clinical judgment” (RWFS) (α = 0.70) and “risk of worsening/complications of the patient’s condition due to flaws in advocacy and delegation” (RWFA) (α = 0.73), both with two items. The “Risk of falls and pressure ulcers” (RFPU) and “Occurrence of falls and pressure ulcers” (OFPU) factors were grouped, giving rise to a single factor of evaluation of the “risk and occurrence of falls and pressure ulcers” (ROFPU), with four items (α = 0.75).

The low internal consistency of the factors UA, ROME and GPS determines the need to confirm this factorial structure through CFA in a larger sample.

The CFA results in the original model^(^ ^4^ ^)^ and in the model resulting from this EFA, in a sample of 685 nurses, are indicative that the latter model fits better to the study sample, in the two subscales, compared to the original one (NP : χ^2^ (49) = 381.34, *p* < 0.05, AE: χ^2^ (0) = 80.74, *p* < 0.05), presenting lower MECVI (NP: 4.34 *vs*. 3.81, AE: 0.69 *vs.* 0.58), thus selecting this factorial structure.

The analysis revealed an acceptable goodness-of-fit, but only fair in the general indices (NP: χ^2^/df = 3.38, CFI = 0.87, GFI = 0.84, RMSEA = 0.06, MECVI = 3.81, AE: χ^2^/df = 4.93, CFI = 0.90, GFI = 0.94, RMSEA = 0.08, MECVI = 0.58).

The λ and λ^2^ were adequate, but the item 7.2 (*adverse events associated with nursing practices could be avoided*) of the GPS dimension had lower values than those previously established (λ = 0.29), and were thus removed from the model.

As for normality, all items presented values considered adequate. However, several observations are considered as multivariate outliers (p_1_ and p_2_ < 0.001). In a conservative strategy, the analysis was reworked excluding eight observations, with high D^2^, with no evidence of improvement in the goodness-of-fit of the subscales, and it was decided to maintain these observations.

**Figure 3:**
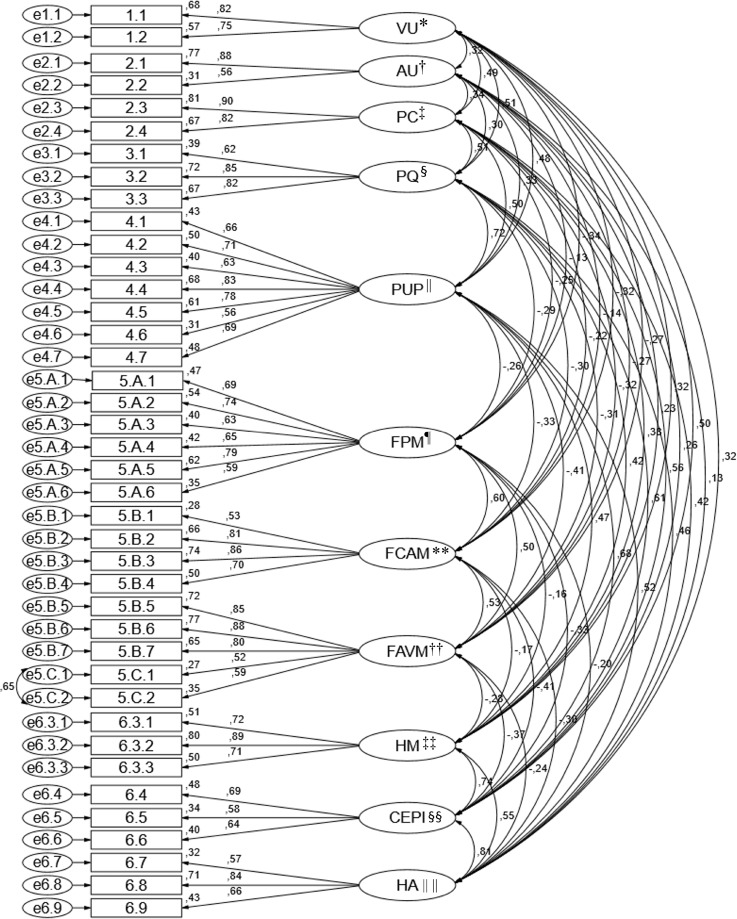
Factorial structure of the refined model of the Nursing Practices subscale of the Scale of Adverse Events associated with Nursing Practices

^*^US - User Surveillance; ^†^UA - User Advocacy; ^‡^PC - Privacy and confidentiality; ^§^FP - Fall Prevention; ^||^PPU - Prevention of Pressure Ulcers; ^¶^MPE - Medication Preparation Errors; ^**^CFAMA - Communication Failure Associated with Medication Administration; ^††^FMAM - Failures in Medication Administration and Monitoring; ^‡‡^HH - Hand hygiene; ^§§^CPPE - Care with Personal Protective Equipment; ^||||^EH - Environmental hygiene

The MI analysis showed a high correlation between the measurement errors of items 5.C.1 (*failures in monitoring the rhythms of infusions*) and 5.C.2 (*failures in monitoring the effects of medication*) (MI = 287.76), belonging to the FMAM factor, which is theoretically justified by the similarity and proximity of the formulation and contents of the items, suggesting the refinement of the model. The solution obtained in the NP subscale, with the correlation of these errors, showed good fit (NP: χ^2^/df = 2.88, CFI = 0.90, GFI = 0.86, RMSEA = 0.05, MECVI = 3.30), according to Figure 3.

**Figure 4:**
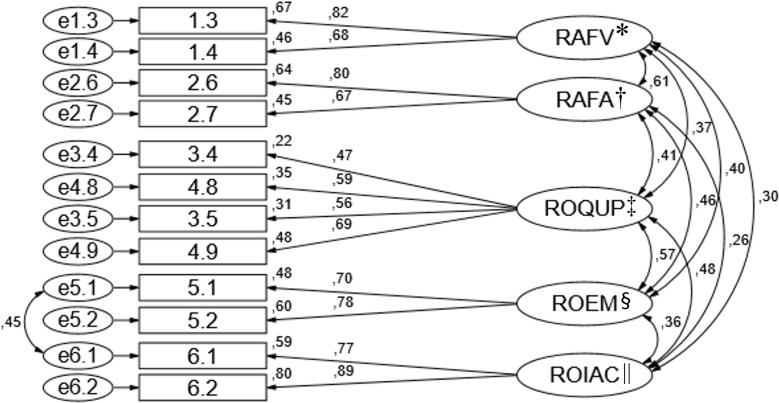
Factorial structure of the refined model of the Adverse Events subscale of the Scale of Adverse Events associated with Nursing Practices

^*^RWFS - Risk of worsening/complications of the patient’s condition due to failures in surveillance and clinical judgment; ^†^RWFA - Risk of worsening/complications of the patient’s condition due to flaws in advocacy and delegation; ^‡^ROFPU - Risk and occurrence of falls and pressure ulcers; ^§^ROME - Risk and occurrence of medication errors; ^||^ROHAI - Risk and occurrence of healthcare-associated infections

In the AE subscale, the internal consistency of the GPS factor (α = 0.43, simultaneously in the EFA and CFA), the factor loading of item 7.2, as well as the fact that it consisted of only two items, justified its removal from the model. MIs also showed a high correlation (MI = 66.59) between the measurement errors of items 5.1 (*there is a risk of occurrence of medication errors*) and 6.1 (*there is a risk of healthcare-associated infections*). Thus, although they belong to different factors, from the theoretical point of view, similarity and proximity are identified, both in the formulation and in the content of the items, proceeding to the refinement of the model. The simplified model, with five factors, showed good fit (AE: χ^2^/df = 4.62, CFI = 0.93, GFI = 0.95, RMSEA = 0.07, MECVI = 0.39), according to Figure 4.

The final refined model fit significantly better than the initial model, in the study sample, in both subscales (NP: χ^2^ (1) = 349.91, p < 0.05, AE: χ^2^ (19) = 106.83, p < 0.05), and the MECVI was also lower (NP: 3.81 *vs.* 3.30; AE: 0.58 *vs.* 0.39).

The construct reliability was adequate in most dimensions (CR and α ≥0.70), with the exception of two factors in the NP subscale (UA and CPPE) and two in the subscale AE (ROFPU and ROME), which present slightly lower according to Table 1. The standardized factor loadings varied in the NP subscale between 0.52 and 0.90, and in the AE subscale between 0.47 and 0.89. The individual reliability of each item varied in the NP subscale between 0.28 and 0.81 and in the AE subscale between 0.22 and 0.80 (Figures 3 and 4).

**Table 1:** Analysis of construct reliability, convergent validity, and discriminant validity of the Factors of the Scale of Adverse Events associated with Nursing Practices (refined model) in a sample of nurses. Central and North Regions, Portugal, 2015

Subscale	Factors	Items	Mean score	CR^*^	α^†^	AVE^‡^	ρ^2§^
Nursing practice	US^||^	2	3.06	0.76	0.76	0.62	0.07 – 0.26
	UA^¶^	2	2.33	0.69	0.66	0.54	0.02 – 0.12
	PC^**^	2	4.25	0.85	0.85	0.74	0.05 – 0.31
	FP^††^	3	4.56	0.81	0.80	0.59	0.08 – 0.52
	PPU^‡‡^	7	2.69	0.87	0.86	0.49	0.07 – 0.52
	MPE^§§^	6	1.68	0.84	0.84	0.47	0.02 – 0.36
	CFAMA^||||^	4	2.22	0.82	0.81	0.54	0.02 – 0.36
	FMAM^¶¶^	5	1.02	0.85	0.86	0.55	0.06 – 0.28
	HH^***^	3	3.25	0.82	0.80	0.60	0.02 – 0.55
	CPPE^†††^	3	3.41	0.68	0.68	0.41	0.11 – 0.65
	EH^‡‡‡^	3	3.57	0.74	0.71	0.49	0.02 – 0.65
Adverse events	RWFS^§§§^	2	1.84	0.72	0.71	0.56	0.09 – 0.38
	RWFA^||||||^	2	2.14	0.70	0.70	0.55	0.07 – 0.38
	ROFPU^¶¶¶^	4	2.25	0.67	0.66	0.34	0.14 – 0.32
	ROME^****^	2	2.16	0.70	0.68	0.54	0.13 – 0.32
	ROHAI^††††^	2	2.66	0.82	0.81	0.69	0.07 – 0.23

^*^CR - Composite reliability; ^†^α-Cronbach’s *alpha*; ^‡^AVE – Average variance extracted; §ρ^2^ - Square of the correlation between factors; ^||^US - User Surveillance; ^¶^UA - User Advocacy; ^**^PC - Privacy and confidentiality; ^††^FP - Fall Prevention; ^‡‡^PPU - Prevention of Pressure Ulcers; ^§§^MPE - Medication Preparation Errors; ^||||^CFAMA - Communication Failure Associated with Medication Administration; ^¶¶^FMAM - Failures in Medication Administration and Monitoring; ^***^HH - Hand Hygiene; ^†††^CPPE - Care with Personal Protective Equipment; ^‡‡‡^EH - Environmental Hygiene; ^§§§^RWFS - Risk of worsening/complications of the patient’s condition due to failures in surveillance and clinical judgment; ^||||||^RWFA - Risk of worsening/complications of the patient’s condition due to flaws in advocacy and delegation; ^¶¶¶^ROFPU - Risk of Falls and Pressure Ulcers; ^****^ROME - Risk and Occurrence of Medication Errors; ^††††^ROHAI - Risk and Occurrence of Healthcare-Associated Infections

With regard to convergent validity, the AVE proved to be adequate in most of the factors, with the exception of the PPU, MPE and EH (NP subscale), which are close to acceptable, being low in the CPPE (NP subscale) and ROFPU (AE subscale). The comparison of the AVE with the squares of the correlation between the factors revealed discriminant validity of the AE subscale and the general NP subscale, except for the correlation of the PPU with FP and CPPE, and the CPPE with HH and EH.

The analysis of the factorial invariance of the model, in two independent samples (test and validation), showed adequate goodness-of-fit indices in the final factorial solution (NP: χ^2^/df = 2.11, CFI = 0.89, GFI = 0, 82; RMSEA = 0.04; MECVI = 5.13; AE: χ^2^ / df = 3.27, CFI = 0.92; GFI = 0.942; RMSEA = 0.06; MECVI = 0.62). There were no statistically significant differences in the overall fit between the two samples when comparing the free model with a constrained model, in relation to the factor loadings, intercepts and covariance of the factors and, in the case of the AE subscale, also the variance/covariance of the errors (NP: λ: Δχ^2^(40) = 45.68; p=0,248; *Intercepts*: Δχ^2^(40) = 28.55; p = 0.912; *Covariance*: Δχ^2^(55) = 71.67; p = 0.065; *Residuals*: Δχ^2^(41) = 67.75; p = 0.005; AE: λ: Δχ^2^(12) = 9,79; p = 0.635; *Intercepts*: Δχ^2^(12) = 13.77; p = 0.316; *Covariance*: Δχ^2^(10) = 17.60; p = 0.062; *Residuals*: Δχ^2^(13) = 16.03; p = 0.248). Thus, in the two samples, strong invariance in the NP subscale was observed, as well as structural invariance in the AE subscale, confirming the stability of this factorial structure.

## Discussion

The present study aimed to contribute to the analysis of psychometric properties, namely factorial structure, validity, reliability and measure invariance of the SAEANP, constituting as evolution of the development of other investigations.

This complements the initial construction and evaluation of the instrument, which gave rise to a revised version of the scale, leading to the need for new psychometric evaluation studies^(^ ^4^ ^)^.

The EFA, followed by CFA and, subsequently, invariance analysis, showed that the SAEANP has adequate psychometric properties.

More specifically, EFA results indicated a factorial structure with 11 dimensions in the NP subscale. The reorganization of MAE and MSE gave rise to the dimensions CFAMA and FMAM, focusing on “communication failures” (CFAMA), in line with scientific evidence, which points out the communication problems between the medical and nursing staff as a factor causing the occurrence of AE, namely medication administration failures^(^ ^20^ ^-^ ^22^ ^)^.

It was also identified the constitution of a new dimension, PC, composed of two new items of the revised version, regarding patients’ privacy and confidentiality, increasing the specificity of the analysis of the instrument in a similar way to an earlier study^(^ ^12^ ^)^. As for item 2.5, this was eliminated because it presented higher saturation in a factor different from the original one (UA), thus conditioning its interpretation. Two previous studies in which this item was eliminated due to a low factor loading^(^ ^12^ ^,^ ^23^ ^)^ were also used to support this decision.

In the AE subscale, we opted for a model with six dimensions, similar to the original model. Differences in the RWFS and RWFA dimensions are evident, making it possible to capture these differences, with a subsequent increase in the instrument’s specificity, similar to an earlier study^(^ ^12^ ^)^. On the other hand, the RFPU and OFPU dimensions were grouped into a single factor, consistent with the other dimensions, which associate the risk perception and the occurrence of AE by type, thus standardizing the analysis method.

It is also pointed out that the factorial solution of the CFA shows a better fit to the characteristics of the study sample compared to the original model^(^ ^4^ ^)^. The MI analysis, supported by the theoretical, semantic and conceptual basis, also allowed the refinement of the model through the correlation of the errors of some items.

The GPS factor was eliminated given its internal consistency and the factor loading of item 7.2. This strategy is also based on the results of previous scale evaluation studies, which also excluded this dimension given the values of internal consistency and/or factor loading of the items, suggesting the analysis of the items as indicators of general perception^(^ ^4^ ^,^ ^12^ ^,^ ^23^ ^)^.

In the NP subscale it was necessary to correlate the measurement errors of items 5.C.1 and 5.C.2, which is theoretically justified by their similarity; both refer to “failures in medication surveillance”, and constitute an autonomous factor in the original version^(^ ^4^ ^)^.

It was also chosen to correlate the errors of items 5.1 and 6.1 because both refer to nurses’ perception of *commitment with patients’ safety*, that is, the risk of occurrence of two types of AE (medication errors and HAIs). It is important to note that, contrary to the “Risk of falls and pressure ulcers”, reflecting essentially the clinical condition of the patient, the “risk of medication errors and HAIs” is particularly associated with the intervention of health professionals, thus justifying the correlation among their errors, although they integrate different factors.

Regarding internal consistency, the EFA results showed low values in UA and ROME factors. However, these are similar to those of the initial evaluation of the instrument (UA: α = 0.51, ROME: α = 0.68)^(^ ^4^ ^)^ and the revised version for the UA factor (α = 0.56)^(^ ^12^ ^)^, being even slightly higher in the present study.

In the CFA, there was adequate internal consistency in most of the subscales; however, slightly lower values in the UA and CPPE dimensions of the NP subscale, and ROFPU and ROME of the AE subscale are recognized. It can be seen that the internal consistency of the UA and ROME dimensions is at the threshold of acceptability. However, there is a higher CR than a previous study in the ROME dimension (FC = 0.63). As for the perception of ROFPU, the same study analyzes them in two independent factors, according to the original version of the scale, also showing threshold values of acceptability (CR: RFPU = 0.70; OFPU = 0.67)^(^ ^23^ ^)^. The small number of constituent items of these dimensions is identified as a factor determining reliability, with only two items being identified. However, although low, some authors report that, in the social sciences, α values of 0.60 may be acceptable, provided the results are interpreted with parsimony^(^ ^24^ ^)^.

Regarding the construct validity, only one item of the AE subscale is identified with a value slightly less than 0.50, conditioning the individual reliability. Some authors consider factor loadings equal to or greater than 0.30 or 0.40 in EFA acceptable in the social sciences^(^ ^18^ ^,^ ^25^ ^)^. However, in the CFA, values lower than 0.50 influence factorial validity and, subsequently, convergent validity, by conditioning the AVE value^(^ ^14^ ^)^. The item 3.4 (There is a risk of falls in patients) (λ = 0.47) conditioned the AVE value in the ROFPU dimension, but for theoretical reasons and due to its importance to guarantee the evaluation of the latent construct of risk of occurrence of falls associated with this dimension, we opted for its maintenance in the model.

The convergent validity was found to be on the threshold of acceptability in the PPU, EH and MPE dimensions, being lower in the CPPE and ROFPU dimensions due to the high variability in the factor loadings of the items. The discriminant validity revealed adequacy in the AE subscale and in the generality of NP, being affected in the PPU, CPPE and EH dimensions.

This work was thus a fundamental contribution to the knowledge of the psychometric properties of SAEANP, complementing the previously elaborated work of construction and initial evaluation of the instrument, which integrates the EFA; in this study, we developed not only the EFA, but also the CFA of the factorial structure of the model and its factorial invariance.

The results show the adequacy of the proposed model to evaluate the nurses’ perception about the AE associated to nursing practices in the hospital context from the perspective of process and results. This is an important tool for promoting health care safety, giving nurses a key role in managing patient risk and safety. The evaluation of the results sensitive to nursing care, namely the AE, aimed at the continuous improvement of the quality and minimization of associated costs for patients and health systems is important. In spite of the limitations found in the validity of the construct, it is worth noting the stability of this factorial solution, proving the strong invariance of the NP subscale and structural invariance of the AE subscale, in two independent samples.

However, the results obtained should be analyzed considering the limitations of the study, namely those related to the reliability of some dimensions, construct validity and type of sampling. It should be noted, however, that although the sample is not random, conditioning the representativeness and generalization of results, it was decided to use a larger sample than the one usually recommended for CFA, so as to adequately translate the population variability and allow the invariance analysis. Due to the limitation of nurses’ voluntary participation, in a convenience sampling process, the maximum number of participants in the target population was included and this contributed to improve the external validity of the results.

It should be noted, however, that the representativeness of the sample was sought. Because the actual values of the target population was unknown, this was determined based on the assumption that their characteristics should not be significantly different from the population of active Portuguese nurses enrolled in the Nurses’ Order (*Ordem dos Enfermeiros*). Regarding gender, at the national level, 81.82% of the nurses are female and 18.18% are male^(^ ^19^ ^)^, and there are no significant differences between the study sample and the Portuguese nurses’ population, although the present sample was not random.

Additional studies with different sample units are still necessary to analyze different factorial structures in order to identify the most appropriate model. It is also suggested that new assessments of the scale be made especially with the inclusion of new items in the generality of dimensions, mainly with regard to “patient advocacy” and “risk and occurrence of adverse events” on a global scale, with the aim of improving its psychometric properties.

## Conclusion

The present study contributed to the evaluation of the psychometric qualities of the SAEANP, an instrument for evaluating the nurses’ perception about the AE associated with nursing care in the hospital setting. The factorial analyses supported the refinement of the original model. The refined model showed good overall fit, confirming its stability and invariance in two independent samples.

The SAEANP is adjusted to assess nurses’ perceptions of the frequency of NP that may prevent AE, as well as the risk and occurrence of AE associated with health care, including nursing care, in the context of hospitalization. However, some limitations were identified regarding construct reliability and validity, and additional studies are needed.

This scale is useful for management as a tool to support decision making, with a view to improving the work processes and, subsequently, the quality of health care and patient safety.

## References

[1] Appelbaum NP, Dow A, Mazmanian PE, Jundt DK, Appelbaum EN (2016). The effects of power, leadership and psychological safety on
resident event reporting. Med Educ.

[2] Wang X, Liu K, You L, Xiang J, Hu H, Zhang L (2014). The relationship between patient safety culture and adverse
events: A questionnaire survey. Int J Nurs Stud.

[3] Paiva MCMS, Popim RC, Melleiro MM, Tronchim DMR, Lima SAM, Juliani CMCM (2014). The reasons of the nursing staff to notify adverse
events. Rev. Latino-Am. Enfermagem.

[4] Castilho A, Parreira PM (2012). Design and assessment of the psychometric properties of an
adverse event perception scale regarding nursing practice. RIE.

[5] Freitas JS, Silva AEBC, Minamisava R, Bezerra ALQ, Sousa MRG (2014). Quality of nursing care and satisfaction of patients attended at
a teaching hospital. Rev. Latino-Am. Enfermagem.

[6] Aiken LH, Sloane DM, Bruyneel L, Van den Heede K, Sermeus W (2013). Nurses’ reports of working conditions and hospital quality of
care in 12 countries in Europe. Int J Nurs Stud.

[7] Van Bogaert P, Timmermans O, Weeks SM, van Heusden D, Wouters K, Franck E (2014). Nursing unit teams matter: Impact of unit-level nurse practice
environment, nurse work characteristics, and burnout on nurse reported job
outcomes, and quality of care, and patient adverse events-A cross-sectional
survey. Int J Nurs Stud.

[8] Cuadros KC, Padilha KG, Toffoletto MC, Henriquez-Roldán C, Canales MAJ (2017). Patient Safety Incidents and Nursing Workload. Rev. Latino-Am. Enfermagem.

[9] Cucolo DF, Perroca MG (2015). Factors involved in the delivery of nursing care. Acta Paul Enferm.

[10] Cattell RB (1978). The scientific use of factor analysis in behavioral and life
sciences.

[11] Westland JC (2010). Lower bounds on sample size in structural equation
modeling. Electron Commer Res Appl.

[12] Castilho AFOM (2014). Eventos adversos nos cuidados de enfermagem ao doente internado:
contributos para a política de segurança.

[13] Marôco J (2007). Análise Estatística - com utilização do SPSS.

[14] Marôco J (2014). Análise de Equações Estruturais: Fundamentos teóricos, Software &
Aplicações.

[15] Hair JF, Babin BJ, Krey N (2017). Covariance-Based Structural Equation Modeling in the Journal of
Advertising: Review and Recommendations. J Advert.

[16] Souza AC, Alexandre NMC, Guirardello EB (2017). Psychometric properties in instruments evaluation of reliability
and validity. Epidemiol. Serv. Saude.

[17] Fornell C, Larcker DF (1981). Evaluating Structural Equation Models with Unobservable Variables
and Measurement Error. J Mark Res.

[18] Matsunaga M (2010). How to Factor-Analyze Your Data Right: Do’s, Don’ts, and
How-To’s. Int J Psychol Res.

[19] Ordem dos Enfermeiros (2015). Dados Estatísticos a 31-12-2014. Ordem dos Enfermeiros.

[20] Gnädinger M, Conen D, Herzig L, Puhan MA, Staehelin A, Zoller M (2017). Medication incidents in primary care medicine: a prospective
study in the Swiss Sentinel Surveillance Network
(Sentinella). BMJ Open.

[21] Bohomol E, Tartali JA (2013). Adverse effects in surgical patients: knowledge of the nursing
professionals. Acta Paul Enferm.

[22] Vanderbilt AA, Pappada SM, Stein H, Harper D, Papadimos TJ (2017). Increasing patient safety with neonates via handoff communication
during delivery: a call for interprofessional health care team training
across GME and CME. Adv Med Educ Pract.

[23] Freitas MJBS (2015). Dotação Segura para a Prática de Enfermagem: Um contributo para a gestão
de unidades de saúde.

[24] Maroco J, Garcia-Marques T (2006). Qual a fiabilidade do alfa de Cronbach? Questões antigas e
soluções modernas?. Laboratório Psicol.

[25] Floyd FJ, Widaman KF (1995). Factor analysis in the development and refinement of clinical
assessment instruments. Psychol Assess.

